# Low participation in cancer screening in India: a scoping review of breast and cervical cancer programs

**DOI:** 10.1186/s12885-025-14859-6

**Published:** 2025-11-07

**Authors:** Jubina Balan Venghateri, Priyansh Nathani, Shreya Goyal, Bhakti Sarang, Harshal Rawtani, Priti Patil, Deepa KV, Nethra Jain, Anita Gadgil, Nobhojit Roy

**Affiliations:** 1 WHO Collaborating Centre for Emergency, Critical and Operative Care, Mumbai, India; 2 Department of Surgery, Terna Medical College & Hospital, New Mumbai, India; 3 Department of Statistics, BARC Hospital, Mumbai, India; 4 Laparoscopic and Robotic surgery, Max Super speciality Hospital, Max Institute of GI, Dwarka, Delhi India; 5 Department of Global Public Health, Karolinska Institutet, Stockholm, 17177 Sweden

**Keywords:** Breast neoplasms, Uterine cervical neoplasms, Early detection of cancer, Diagnostic screening programs, India

## Abstract

**Background:**

India is witnessing a high and rising burden of breast and cervical cancers. More than one-third of cases in India are attributed to these two cancers. Early detection and access to affordable and timely treatment are known to reduce the burden of cancer-related deaths. Low and Middle-Income Countries (LMICs) face significant challenges in implementing organized early-detection programs due to inadequate resources, contributing to high mortality from these cancers. Recognizing this critical public health issue, this study evaluates the published literature and government reports on the implementation of breast and cervical cancer screening programs in India.

**Methods:**

Literature was systematically searched from six databases: PubMed, Embase, Scopus, CINAHL, Web of Science, and Google Scholar. In addition, reports on the National Health Mission website were reviewed to capture screening efforts that were not published in the peer-reviewed literature.

**Results:**

59 peer-reviewed manuscripts were identified, from 57 screening programs. The number of screening programs from Northern and Eastern states is low. Community programs focused on task shifting and engagement of local stakeholders for increasing participation. Clinical Breast Examination (CBE) and Visual inspection of the cervix (VIA) remain the mainstay of screening efforts. The main barriers to screening uptake by women were lower education, lower socioeconomic status, lack of transportation, and suboptimal services. Information on screening programs lacked uniformity in reporting and data collection.

**Conclusion:**

These results highlight that screening efforts in India remain disjointed and programs by different agencies need to be aligned through uniform distribution, methodology, and reporting, towards goals set by global initiatives.

**Supplementary Information:**

The online version contains supplementary material available at 10.1186/s12885-025-14859-6.

## Introduction

More than one-third of cancer cases in India are attributed to oral, breast, and cervical cancers [1, 2]. In India, breast and cervical cancers together account for nearly 39% of all cancers among women [3]. According to GLOBOCAN 2020 data, India reported an estimated 178,361 new cases and 90,408 deaths due to breast cancer, and 123,907 new cases and 77,348 deaths due to cervical cancer [4]. Over the past two decades, cervical cancer incidence has shown a steady decline, particularly in urban regions, largely due to improved hygiene and opportunistic screening [5, 6]. Notably, cervical cancer disproportionately affects older women in rural areas, typically aged 40–60 years, due to poor access to healthcare and screening services [7]. Mortality for cervical cancer has declined modestly, while breast cancer mortality remains high, particularly due to late-stage diagnoses [4]. In contrast, breast cancer incidence has been steadily rising, especially among younger, urban women aged 30–50 years, with rates increasing by over 39% between 2012 and 2020 in some regions [1, 6]. Collectively, factors contributing to these trends include changes in reproductive behavior, lifestyle factors, lack of awareness, limited access to screening services, and inadequate infrastructure for early diagnosis and treatment [8, 9].

Early detection and access to affordable and timely treatment are known to reduce the burden of cancer-related deaths in Low and Middle-Income Countries (LMICs) [10]. Organized screening programs have contributed to reduced mortality from these cancers in High-income countries (HICs) [11, 12]. However, screening for cancers in India is still mainly opportunistic, and as a result majority of cancers are diagnosed at the advanced stage [13]. The Ministry of Health and Family Welfare (MoHFW) of the Government of India launched an operational framework for a national screening program for breast, cervical, and oral cancer to roll out for a population of 1.3 billion in 2016 [14]. The suggested strategy for screening was Clinical Breast Examination, Visual inspection with acetic acid application, and oral visual examination, respectively, for breast, cervix, and oral cancers. Despite these efforts, cancer screening programs across India experience low participation [15, 16].

Since health as a subject falls under the jurisdiction of individual states, each state develops and implements its programs by the guidelines established by the central government [17]. Additionally, various regional cancer centers, tertiary care centers, and non-government organizations contribute to screening by implementing district-wide public health programs and research projects. According to the National Family Health Survey-5 (NFHS-5), the overall uptake of cervical and breast cancer screening in India is less than 2% [16]. It is crucial to identify where these programs are conducted and how they operate to understand the current landscape of screening for breast and cervical cancers in India. Since the launch of the mobile platform technology rolled out in India in 2016, it has become imperative to track the progress made in cancer screening programs [18, 19]. However, there is no comprehensive or single source of information on these various cancer screening efforts that function*in silos*. Additionally, addressing the major gaps associated with the current breast and cervical cancer screening efforts is essential. Hence, this scoping review aims to document various breast and cervical cancer screening programs from the peer-reviewed literature as well as government reports. We also aim to appraise the methods of outreach, screening tests used, reasons for low uptake of screening, and their probable solutions described in these programs, with the goal of informing future policies and program development.

## Methods

### Search strategy

This scoping review adhered to the Preferred Reporting Items for Systematic Reviews and Meta-Analysis (PRISMA) reporting guidelines [20]. For this study, we performed an electronic search in six databases: PubMed, Embase, Scopus, CINAHL, Web of Science, and Google Scholar. Briefly, we used the terms *Breast and cervical cancer. *Early detection of cancer, *Cancer screening, and *India and combined them with Boolean operators (e.g., AND, OR, NOT) to perform a comprehensive search for this study. A detailed search strategy customized for each database to give a maximum output of articles is detailed in Supplementary Table (1) A total of 59 articles included in this review are listed in Supplementary Table (2) The search was restricted to studies conducted in India between 2016 and 2023. Although the search was designed to include studies published between 2016 and 2023 to align with the launch of the national cancer screening program in India, we observed that the database algorithms occasionally retrieved studies published in 2015 despite applying filters for 2016 onwards. This discrepancy may be due to variations in indexing or online publication dates. After manual screening, five studies published in 2015 [21–25] were found to be highly relevant to the review objectives and were therefore included in the final analysis.

The peer-reviewed studies mainly reported the efforts of screening documented through research projects and analyzed established screening methods, barriers, and facilitators (Table 2), and newer screening approaches in a research setting. Government reports documented screening coverage and sometimes screening and positivity rates, and targets to be achieved. These parallel efforts are documented separately and not integrated. Hence, we searched both the published literature as well as the National Health Mission (NHM) website and statewide government websites. These sites were visited to capture efforts through National Health Mission (NHM), not published as peer-reviewed manuscripts. These are summarized in Supplementary Table 3.

### Inclusion criteria used for the study

Original published research articles using primary data related to breast and cervical cancer screening and early detection programs but not exclusively screening these cancers were included. We included full-text available studies published in a peer-reviewed English- language journal. Studies conducted in India by any government health facilities, regional cancer centers, medical colleges, and ‘for profit’ and ‘not for profit’ or faith-based non-government organizations were included. Studies describing various screening and early detection methods such as clinical breast examination/mammography, VIA, HPV testing, and colposcopy were included. Studies conducted at the population level/tertiary centers were included.

### Exclusion criteria used for the study

**Fig. 1:**
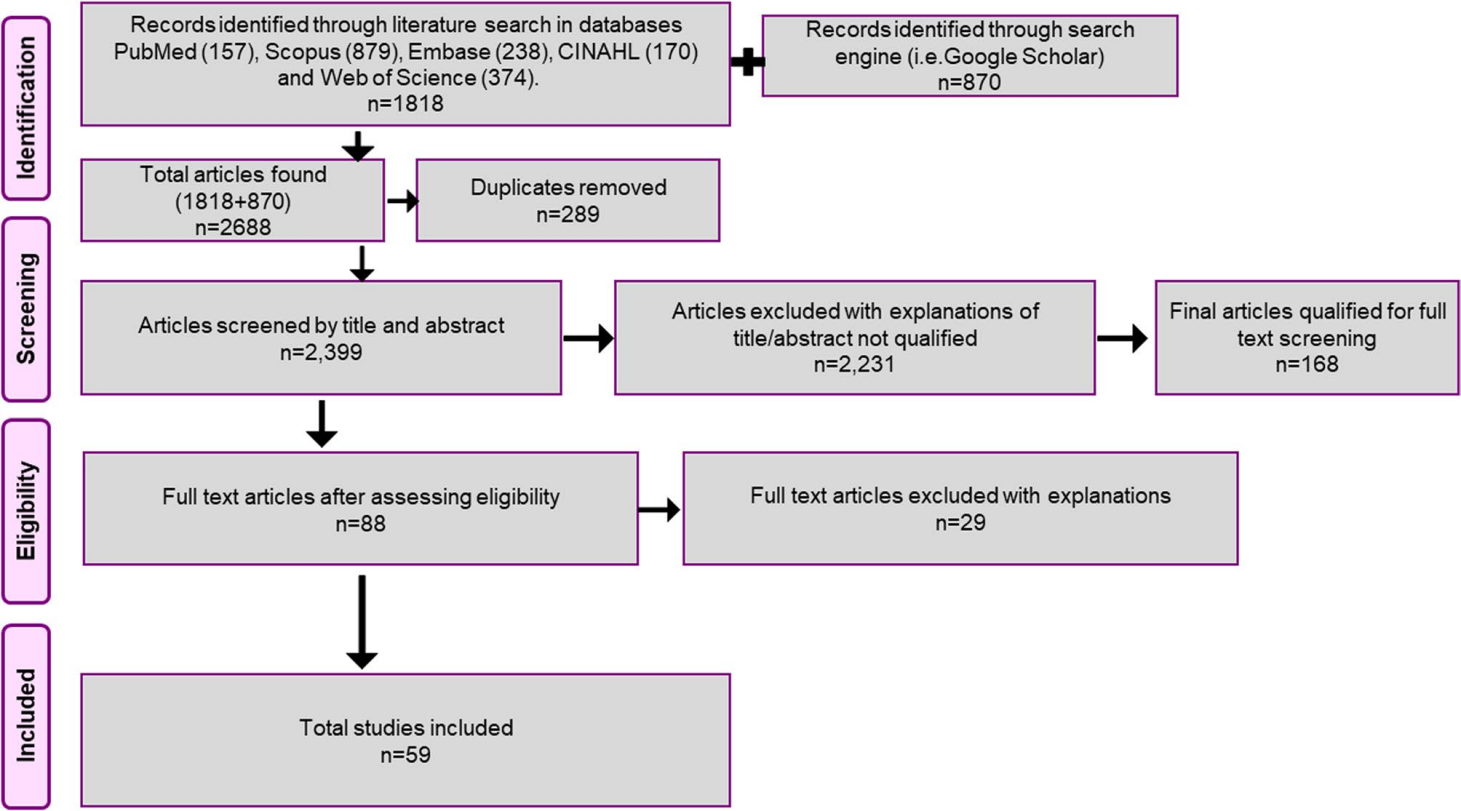
District-wise breast **A** and cervical cancer screening programs conducted **B** in India are highlighted in lighter colors. Missing data from the unreported government-based ‘National Programme for Prevention and Control of Cancer, Diabetes, Cardiovascular Diseases, and Stroke’ and other state-based initiatives undermine the actual number of active cervical cancer screening programs in each state. Many districts are incompletely covered. Studies often focus on cities/villages within the districts or use convenience sampling, and thereby, the map may present an overly optimistic scenario. Two cervical cancer screening studies cited unspecified areas where author affiliations were utilized to identify districts. Ten cervical cancer screening studies span multiple districts, inflating the district count beyond the actual number of programs

Studies related to screening and early detection programs other than breast and cervical cancers were excluded. Literature reviews, commentaries, and non-research studies involving secondary data were excluded. Studies describing the effects of treatment modalities like surgery, radiotherapy, chemotherapy, and genetic studies were excluded. Studies discussing knowledge, attitude, and practice (KAP) of health workers or any population without a description of the screening program or elaborating on screening results were excluded (See Fig. 1). All articles retrieved, using the above criteria, were then uploaded to Rayyan for further screening [26]. Four team pairs comprising 1). JBV & HR, 2). AS & DKV, 3). PN & SG, 4). BS & NJ completed the title-abstract screening. In addition, a third reviewer (AG) resolved the conflicts between the team pairs. All disagreements were resolved through discussions in weekly meetings. The full-text screening was completed by JBV, BS, HR, SG, DKV, and NJ. Any conflict was resolved by the third reviewer AG. We contacted the respective authors for unavailable full-text articles and received responses from only three authors with full-text studies. These were eventually excluded as they were not eligible for inclusion.

## Data extraction and synthesis

Data extraction was independently conducted by seven reviewers (JBV, PN, SG, BS, HR, NJ, and DKV) and then verified by two additional reviewers (AG and PP). A predefined Excel-based tool was used to analyze the extracted data. The state government-driven National Program for Prevention and Control of Non-Communicable Diseases (NP-NCD) was visited to extract data on screening coverage, the planned targets, and the progress of the programs through their Program Implementation Plans (PIPs) and Recordings of Proceedings (ROPs) reports, which are mandated by the NHM for each state. The latest documents for each state and union territories (UT) as available were accessed on 4th September 2024 [27]. Additionally, multiple articles reporting on the same screening program were merged during data extraction to avoid redundancy in program counts.

### Statistical analysis

A descriptive statistical analysis was performed to assess the uptake and progress of breast and cervical cancer screening programs in India since 2016. Screening data were extracted and summarized using summary statistics and frequency distributions. The results were organized and presented using Microsoft Excel 2019 and visualized through tables and graphs to offer a detailed overview of the findings.

## Results

### Characteristics of the included studies

**Fig. 2:**
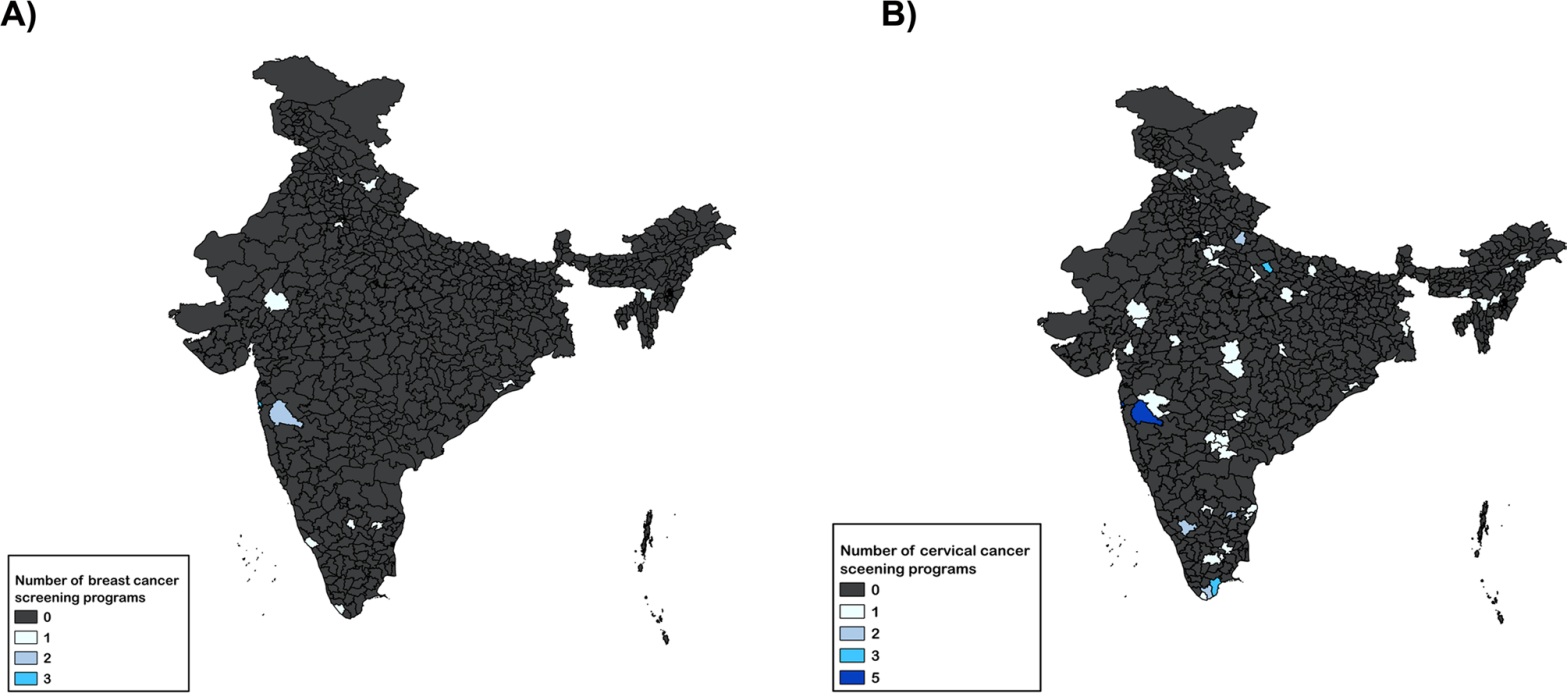
PRISMA flowchart illustrating the stages of the review, and the number of included studies for this study is shown

2688 articles were retrieved from the databases and included in title and abstract screening. Full text was available for 144 of the screened articles. Fifty-nine published articles describing 57 screening programs were included after the full text screening for data extraction. Figure 2 shows the flowchart of the article selection. Among these included articles, 43 studies described cervical cancer programs, nine described breast cancer screening, and seven described both breast and cervical cancer screening programs. Clinical breast examination was the most common method used for breast screening (81%), while Visual Inspection with Acetic Acid (VIA) (48%) and Pap smear (38%) were the common methods documented for cervical screening. 40% of the studies were implemented in rural settings, and one of them included tribal populations. Table 1 describes the characteristics of the included studies. There was a significant disparity in the geographical distribution of cancer screening studies in India. Maharashtra reported 15 (23.4%) of the total studies, followed by Delhi 8 (12.5%), Tamil Nadu 6 (9.4%), and Karnataka 5 (7.8%). Uttar Pradesh, Rajasthan, and Kerala, each reported 2 (3.1%) studies. States with 1 (1.6% each) study include Madhya Pradesh, Orissa, and Manipur, among others.

**Table 1:** Characteristics of the included articles in this scoping review

Characteristics of the included articles	*No. of articles *n* (%)
Cancer Type studied	
Cervical	43 (72%)
Breast	9 (14%)
Both	7 (14%)
Study Design	
Prospective	56 (95%)
Retrospective	3 (5%)
Study Type	
Cross sectional study	44 (73%)
Cohort Study	11 (19%)
RCT	4 (7%)
Research Approach	
Quantitative	45 (76%)
Qualitative	4 (6%)
Mixed	10 (16%)
Study Setting	
Rural + tribal	24 (40%)
Urban	25 (42%)
Mixed (Urban & Rural)	10 (17%)
Sample size	
< 1000	23 (39%)
> 10,000	21 (36%)
1000-10,000	15 (25%)
Screening method for Breast (*n* = 16)	
CBE	13 (81%)
Mammography	2 (12%)
Handheld optical device	1 (6%)
Screening method for Cervix (*n* = 50)	
VIA	24 (48%)
Pap Smear	19 (38%)
HPV Testing	7 (14%)
Funding (*n* = 46)	
Yes	34 (74%)
No	12 (26%)
Screening Program Type	
Government	29 (49%)
Private/NGO	30 (51%)
Outreach strategy	
Population-Based	27 (46%)
Hospital-Based	18 (31%)
Mixed	14 (23%)

Abbreviations RCT – Randomized Controlled Trial, CBE–Clinical Breast Examination, VIA – Visual Inspection with Acetic Acid, HPV – Human Papillomavirus, NGO – Non-Governmental Organization

*The total in each category exceeds the total number of articles analyzed, as some programs included more than one category/option

### Strategies for cancer screening programs

Task shifting to CHWs or nurses for examining individuals at risk/symptomatic individuals has been the common strategy documented by 40% of manuscripts for breast as well as cervical cancers. All programs emphasized the feasibility and need of integrating cancer screening with existing health services in rural settings and the critical role of systematic follow-up [21, 28–31]. Most studies focused on increasing demand and uptake of screening by the community. However, 15 of the 57 (26.3%) programs focused on supplying or providing innovative approaches and feasible solutions to improve screening at the community level.

### Methods for screening and early detection

Most of the studies used CBE, VIA, and OVE for screening for breast, cervical, and oral cancers, respectively. Three programs on HPV testing by self-sampling, two programs on point of care use of smart colposcope, and imaging breast screening device like iBreastExam (iBE) a hand-held non-invasive, radiation-free device that uses piezoelectric sensor based technology to detect tissue stiffness and identify potential abnormalities in breast tissue [32, 33]. This has been used to detect breast lumps as an adjunct to clinical breast examination. One program described liquid-based cervical cytology. All but one of these newer approaches were described for cervical cancer, except one that used a handheld imaging device for early detection of breast cancer. Details of the screening methods used are given below.

#### Cervical cancer

43 of the included 59 studies described cervical cancer screening. Seven described cervical cancer screening along with breast cancer and/or oral cancers. Though most studies included the general population, one study each included prison inmates, police personnel, and women participating in trade fairs. 24/59 (40%) studies included tribal/rural women exclusively. Cervical cancer screening in Women living with HIV was addressed by three studies [34–36].

##### Visual Inspection with Acetic Acid

Among the 50 studies that implemented cervical cancer screening, VIA was the most used test 24 (48%) for screening. This was a method of choice in camps, mobile clinics, and hospital-based settings. VIA positivity rates ranged from 2.8 to 28% in population-based studies. The sensitivity and specificity of naked eye examination after the application of the VIA test were 90% and 39.5% respectively. Positive Predictive Value (PPV) was 43.9% and the Negative Predictive value (NPV) was 88.2% [37, 38]. Sensitivity for the VIA test increased to 100% when 6X magnification was used instead of a naked eye examination.

##### Human papillomavirus test

Seven studies used HPV sampling for cervical cancer screening. This was implemented both in the community and in hospital-based screening initiatives. HPV positivity ranged from 3.2 to 12.6% [39–41]. Self-sampling vs. HCW collected samples performed equally well for HPV screening. The acceptability of the Self-test was higher than the CHW collected samples [40]. The sensitivity and specificity of HPV test from menstrual pad samples were 83% and 99% respectively [42] when compared with standard HPV collection.

##### PAP test

19 studies implemented PAP alone or in combination with VIA and HPV testing as screening methods. Reported PAP positivity ranged from 3.7 to 15.8% in the community setting. Gynaecology departments of tertiary care centers reported higher PAP positivity rates ranging from symptomatic women 50–96% [29, 43, 44]. PAP was more commonly used in hospital settings with the availability of cytology services.

Novel approaches in cervical screening included the use of menstrual pads for HPV sampling [42], the use of folate receptor-mediated staining solution detection [38], and smart scope [37] for identifying cervical abnormalities. Women living with HIV were the focus of cervical cancer screening in 3 studies [36, 45].

High positivity rates of 35%, 37% and 23% were seen for VIA, HPV, and PAP respectively, in these women; hence screen and treat approach was implemented [34, 46] in some screening camps. A change to screen, test, and treat was needed due to the refusal from patients to get treated at the same visit [23]. The main reasons for refusal of treatment in the same visit were the need for a second opinion (doctor/family members) and concurrence from the family or husband for further procedures [47]. Providers deferred further procedures when they were unsure about VIA positivity. Due to this low treatment rate, it was switched to the “Screen, test, and treat” protocol [48].

#### Breast caner

All breast cancer screening initiatives utilized community educational activities along with clinical breast examination as a screening and early detection strategy. One study used digital mammography [49]. One study implemented an ‘*I breast*’ examination for screening [31]. CBE and ‘*I breast’* performances were comparable. CBE showed sensitivity, specificity, PPV, and NPV of 65%, 94%, 52%, and 96%, respectively. ‘*iBreastExam’ *had sensitivity, specificity, PPV, and NPV of 84%, 94%, 60% and 98%, respectively [31]. Digital mammography was used in the hospital setting combined with screening in camps conducted in the population. Screen positivity rates were 20% for digital mammography [49]. CBE screen positivity results varied from 0.55 to 15% in the literature, and cancer pick-up rates varied from 0.02 to 2.8% in the population-based screening programs [50, 51].

### Community outreach efforts

Out of 59 studies, 27 (46%) were population-based, 18 (31%) were hospital-based, and 14 (23%) combined both population-based and hospital-based approaches. The hospital-based programs relied on convenience sampling and examined individuals reporting to gynaecology departments for various symptoms or for screening. Community-based studies engaged primary healthcare workers, community representatives, and Accredited Social Health Activists (ASHA) community health workers) to generate awareness or to actively engage with the community before bringing them to the hospital or screening camps [30, 49]. Other awareness strategies included radio talks, workshops, seminars, brochures, newsletters, letters of invitation, and audio-visual aids [21, 24, 52–56].

Population-based screening programs were carried out at multiple public spaces such as PHCs and sub-centers (screen and treat paper [39, 57], NCD clinics [58], rural health training centers [59], government schools [48, 60, 61], village satellite clinics, or common community buildings [19, 39, 62, 63], trade fairs [58], religious centers [62, 63], political party offices [62, 63], Gram Panchayat offices [61], and Anganwadi centers [48, 61], ensuring wider community coverage. Regular screening camps were held, often in collaboration with local NGOs, enhancing the workforce and providing necessary resources [39, 45, 56, 64, 65]. Local leaders participated by mapping areas for targeted interventions, facilitating household surveys, and promoting screening camps [39, 40, 62, 63, 66, 67]. In some regions, home-to-home visits were conducted for data collection and identification of screen-eligible populations, directly engaging [38, 42, 47, 50, 67, 68].

Mobile screening vans provided a wider reach [34]. Three studies implemented mobile clinics for screening. Two studies were conducted in urban areas of, Pune [34] and Mumbai (TMH) [66] and 1 in rural India [48], Mysore. Two programs exclusively used the van for cervical cancer screening whereas 1 unit used in Mumbai screened 3 common cancers oral, breast and cervix [66] (TMH study Mumbai). Several studies emphasized the importance of repeated follow-up camps at the same location or in extending camps beyond regular working timings into late evenings to encourage participation and effective follow-ups [56, 62, 63]. The mHealth prototype was explored to streamline data collection, improve reporting accuracy, reduce errors, and enhance screening coordination [19]. CHWs found it more reliable and efficient than paper-based systems, boosting confidence and efficiency, patients saw the mobile tools as a sign of the hospital’s commitment to their care, fostering trust [19].

### Uptake of breast & cervical cancer screening uptake

Nine of the 50 (18%) studies documented the percentage of cervical cancer screening uptake, whereas only four of the 8 (44.5%) studies mentioned breast cancer screening uptake. The indicators of participation, performance of the program, and cancer detection rates were not uniformly reported and hence could not be compared between programs and regions. Uptake in cervical cancer screening programs ranged between 9 and 93%, with most studies having an uptake percentage between 40 and 60% [39, 48, 55, 60, 61, 65, 69]. The breast cancer screening uptake ranged from 10 to 94% with most studies documenting an uptake of more than 60% [50, 53, 54, 63]. The included studies highlighted that the uptake of screening increased after education and awareness activities. However, despite these awareness initiatives, the uptake of cervical cancer screening remained relatively low, which was attributed to fear [70–73], apprehension, and the slightly invasive nature of the screening tests by the reviewed studies [70, 72–74] (Table 2).

**Table 2:** Facilitators and barriers of screening uptake observed in this study are listed

Category	Facilitators	Barriers
Community level factors	Young age, family history of cancer, education, and religion [62, 63]. Higher Socio economic status [24].	Lower education [24, 44, 49, 64, 75–78]. Fear of examination, myths, Lack of awareness and lack of autonomy to make health related decisions [22, 68, 77].
Health Systems	Insurance coverage facilitated [62, 63] Financial incentives [47, 52]. Low-cost VILI/VIA, presence of trained staff at all levels of care, standardized protocol, structures IEC, utilization of specialist doctors in the network, communication with community-based civil groups [79]. Community engagement task shifting [48].	Lack of infrastructure, distance from facility [48], inadequate training for the CHWs [29]. Poor quality of services available [22, 68, 77].

Abbreviations used CHWs – Community Health Workers, IEC – Information, Education, and Communication, VIA – Visual Inspection with Acetic Acid, VILI – Visual Inspection with Lugol’s Iodine

### Reporting of cancer screening implemented by state governments

National Programme for Prevention and Control of Non-Communicable Diseases NP-NCD programs by various states report their progress to the National Health Mission of the Government of India government of India. Health being a subject under state government jurisdiction, these reports were accessed through the national health mission webpage link of various states and union territories [27]. Coverage rates are currently alarmingly low, and PIPs (Program Implementation Plans) and ROPs (Recordings of Proceedings) are incomplete and inconsistent. Different states and UTs use heterogeneous metrics for reporting, as data is reported under “*Achievement and Targets*,” “*Progress*,” “*Likely Achievement*,” or “*Proportional Target Achieved*,“. Interpretation, monitoring, and comparison of these data are hence, challenging. Terminologies used to describe key metrics are inconsistent. “The population to be screened”, the “aimed coverage or target”, and the “actual population covered” or screened within a specific time frame are heterogeneous and interchangeably used. Furthermore, many reports fail to distinguish between screenings for oral, breast, and cervical cancers, leading to gaps in comprehensive reporting. (Supplementary Table 3). The reports from state government websites of Andhra Pradesh, Kerala, and Karnataka provided examples of comprehensive data reporting and initiatives to improve participation. Reports from Andhra Pradesh included clear targets and achievement rates and actual population count screened in absolute numbers for oral, breast, and cervical cancer screenings, with a 78% success rate for each. The government of Kerala has launched a live cancer screening dashboard as part of its Cancer Control Programme, maintained by the e-health project management unit, Department of Health and Family Welfare, Government of Kerala [80]. In Karnataka, a new doorstep screening initiative for oral and breast cancers, integrated with NCD management, is awaiting State Cabinet approval. Meanwhile, the Assam Cancer Care Foundation is setting up ‘Swasth Assam’ kiosks in medical college OPDs to enhance NCD screening and health awareness among patients and their attendants.

## Discussion

This scoping review analyses 59 peer-reviewed articles documenting 57 breast or cervical cancer screening programs in India that were implemented by public and private organizations in the last 10 years. Breast and cervical cancer screening programs were predominantly documented from the southern states of the country, with very few programs from Northern, Northeastern, and central India. Task shifting to community health workers and community involvement in creating awareness prior to screening was the main strategy implemented in population-based programs to increase the demand of screening. Very few programs addressed health system-level innovations and solutions to provide more effective screening. CBE and VIA were the most used tools, with some emerging evidence suggesting good acceptance of HPV testing by women, especially when self-sampling was implemented. Data reported by the State government websites were heterogeneous and lacked uniform indicators.

###  Geographical distribution of screening programs

Despite the implementation of free nationwide screening, we observed that very few breast and cervical cancer screening programs exist and are published in peer-reviewed literature from India. The majority of the programs are concentrated in the southern and western parts of the country, especially in the states with high GDPs [59, 61]. The cancer incidence is rapidly rising in the Northeastern states of India, but there are very few programs for cancer screening in the area [81].

Various states in India are at different levels of epidemiological and demographic transition. The health agenda of many states, like in the aspirational states of Bihar, Orissa, and Madhya Pradesh, the health agenda is driven by high maternal and infant mortality figures. They prioritize maternal and childcare programs and infectious diseases, often leaving behind cancer control efforts. States with higher literacy rates, lower maternal mortality, and better health indicators reported more screening programs. The uptake of government run screening programs is higher in these states, as documented by the fifth round of the National Family Health Survey (NFHS5) [15] (Supplementary Table 2). Screening being lower priority in low and middle income countries, as seen by the absence of organized screening programs in various Asian as well as African countries [82–85]. A systematic review of screening programs for breast and cervical cancer screening by Srinath et al. has documented similar challenges in prioritizing screening programs in other low and middle- income countries [86]. They similarly prioritize infectious diseases, maternal and child health care. India, being a large country, it is said that various states reflect ‘countries within a country’ [87].

### Community engagement

Community-based screening programs documented that awareness campaigns by local community leaders, village chieftains, and other members increased the participation of women in screening programs [22, 55, 68, 77]. Reviewed studies also documented that husband’s preferences and priorities determined the health-seeking in women due to a lack of autonomy to make decisions regarding their health [42, 47]. These findings suggest that family and community-level barriers need to be mitigated through the involvement of larger groups of stakeholders like families, local leaders, or religious leaders. Autonomy of women in making healthcare-related decisions was also explored by Idris Edayu et al. in their systematic review [88]. They found that the education of women, their occupation, their husband’s occupation, and the residence was similarly shown to affect all healthcare related decisions. These findings were similar to the findings in the studies included by our scoping review [88]. Scoping review of community engagement in cervical cancer screening programs from Sub-Saharan Africa by Habila et al. has revealed similar factors to promote screening. They have advocated inclusion of larger stakeholder groups like villagers, religious leaders, traditional leaders, educational institutes to be involved in the screening efforts [83].

###  Health system level initiatives to facilitate screening uptake and provision

India follows a mixed healthcare financing model with public spending at 3.8% of GDP (2022) and out-of-pocket expenditure (OOPE) still comprising 47.1% of total health spending [89]. While public schemes like Ayushman Bharat (PM-JAY) cover hospitalizations and some diagnostics, population-based breast and cervical cancer screening is not uniformly included, often relying on government or NGO initiatives. A range of non-governmental organizations (NGOs) in India have demonstrated that context-sensitive, community-anchored models can meaningfully advance cancer screening, especially in underserved urban and rural populations. Notable examples include Sahaya Hastha Trust (Karnataka), Indian Cancer Society (Delhi and South India), Maina Foundation in collaboration with All India Institute of Medical Sciences (AIIMS) Rishikesh (Uttarakhand), World Healthal Trust and DLF Foundation (Haryana), Institute for Rural Health Studies (Andhra Pradesh), Population Services International (Uttar Pradesh), and Prayas (Maharashtra) and the North East Cancer Foundation Trust (NECFT), which works extensively in Assam and neighbouring states to increase awareness, screening, and referral for cancers prevalent in the region [90–92].

Their screening strategies typically involved community-based or mobile screening camps, walk-in centers, and ASHA/ANM-led outreach, using tools such as Pap smears, VIA, CBE, and HPV DNA testing, often alongside structured awareness campaigns. Many programs employed task-shifting, training nurses and midwives in VIA, thermal ablation, and basic diagnostics, and partnered with tertiary institutions like AIIMS, Kidwai Memorial Institute of Oncology, MNJ Regional Cancer Centre, and Apollo Hospitals for referral support. Several key lessons emerge here, such as the effectiveness of low-cost, scalable ‘screen-and-treat’ approaches, the feasibility of task-shifting in resource-constrained settings, and the importance of community mobilization and digital tools (e.g., WhatsApp) for coordination. However, post-screening follow-up remains a significant challenge, highlighting the need for better continuity of care. The cancer screening programs in India are implemented at the primary healthcare level for free of cost by various state governments. This helps in taking the screening closer to peoples’ homes. Despite these provisions, improving participation still remains a challenge. In the private sector, screening costs vary: VIA and CBE cost INR 100–300 (~$1–4), Pap smears INR 200–1,500 (~$3–18), and HPV DNA testing or mammography over INR 2,000 (~$24) [93–95]. Thus, even modest screening costs may represent a significant financial burden for many households, potentially limiting access and uptake. Thus, even modest screening costs may represent a significant financial burden for many households, potentially limiting access and uptake. Increasing cancer awareness, as well as making people aware that treatment will be financed by the government under its health insurance scheme called Pradhan Mantri Jan Arogya Yojana (PMJAY) and similar state level financing initiatives, is important to improve screening uptake.

Many other system-level initiatives were seen to increase screening uptake, like providing low-cost and accessible screening methods, such as Visual Inspection with Lugol’s Iodine (VILI) or Acetic Acid (VIA) and ensuring trained staff and standardized protocols at all levels of care [79]. These methods eliminate the need for the presence of a specialist at the screening site and screening can be conducted at the community level. Limited infrastructure and the distance to healthcare facilities make access difficult for many, especially in rural areas. These barriers were documented to suggest taking cancer care closer to people’s homes to increase coverage [48]. Commonly cited barriers to screening uptake for which system-level solutions are needed were financing the screening programs, outreach of programs to remote and rural areas, cancer awareness, provision of culturally suitable screening methods by women’s healthcare providers. WHO’s health system strengthening six-block framework was used by Gravitt et al. to underline the importance of a health systems lens to strengthen screening programs. They documented that though health systems approach should be used by policy makers while implementing programs, contextual factors affect implementation, and early stakeholder engagement is advocated [96]. Our study documented that 51% of the studies were undertaken by private or non-government providers. These services were mainly hospital-based in the medical colleges or through NGOs under privately funded project settings [97].

Various studies have emphasized public-private partnerships (PPP), especially in limited resource settings, for the provision of care at the primary health care center level. Joudyian et al. in their scoping review have concluded that PPP can facilitate access to health care services, especially in remote areas [98]. Governments should consider long-term plans and sustainable policies to start PPPs in PHC and should not ignore local needs and context [98].

###  Task shifting

Most of the screening programs analyzed were conducted by nurses and other paramedics through task sharing and task shifting. Task sharing/shifting has been an important aspect of screening strategies for cancers in other LMICs to improve access and uptake of services in the existing limited workforce. Six countries in sub-Saharan Africa, Kenya, Nigeria, Tanzania, Uganda, Zambia, and Zimbabwe, integrated cervical cancer screening with family planning services and HIV/AIDS programs. This achieved higher utilization of task-shifted healthcare workers and other resources, along with increased uptake of all the programs [99, 100]. Task shifting has also been used successfully in global surgery in African countries where a lack of qualified surgeons leads to a lack of access to essential surgeries like cesarean sections, hernias, and laparotomy. Competency-based credentialing has been successfully implemented to bridge this gap by many Non-Government Organizations with support from the local governments. Various cadres of healthcare workers have been trained, supervised, and then certified to perform various tasks beyond their regular duties [101]. Lessons can be learned from these strategies to implement task sharing and credentialing at various levels of cancer control strategies, including screening. Two of the reviewed studies documented that community health workers (CHWs) were trained to perform screening for common cancers at people’s homes. Certifying a task-shifted workforce can improve access to screening services and potentially participation [41].

### Use of innovative approaches and technologies

While most articles focused on increasing demand for screening by the population, there was little focus on improved and low-cost provision of services. Research on innovative approaches to provision beyond task shifting was lacking. Some of the South American countries used low-cost rapid HPV testing through self-sampling as a more acceptable method of triage, and a quarter of a million women got screened in 3 years, with coverage reaching above 85%. This bypassed pelvic examination, the availability of skilled workers, clinic access, and discomfort, leading to higher coverage [102]. HPV testing as a tool was addressed in studies by [21], but more such studies on a larger scale are needed to understand the cost of implementing such a program. Kenya similarly focused on improving diagnostic and treatment facilities in the first phase of the cancer control program before increasing the demand for screening by implementing population-based screening programs [103, 104]. None of the programs focused on improving diagnostic care or referral pathways prior to screening initiatives. None of the reviewed articles piloted the use of portable ultrasound as a point-of-care triaging tool to detect breast cancers at an early stage. Ultrasound is low-cost, radiation-free, portable, and available and can be used to triage by distinguishing between a cystic and a solid mass [105]. Similarly, none of the articles discussed the use of patient navigators as recommended by the global breast cancer initiative [105]. Low and middle-income countries where low education in women is a great challenge to follow up and completion of diagnostic tests and treatments, navigator assistance through the journey of cancer care can prove valuable and needs to be explored. There was a lack of focus on studies attempting to reduce or optimize the cost of screening. Kenya’s model of strengthening diagnostic level facilities and referrals prior to screening programs needs to be tested in India [104].

### Heterogeneity in reporting screening data

Data on coverage of the population, participation from the community, and performance of the program were not uniformly documented in the peer-reviewed articles, as well as in various state government portals. None of the studies documented the use of these indicators to strengthen the programs. National Cancer Registry Program, as well as Reproductive, Maternal, New-born, Child and Adolescent Health (RMNCH + A) programs in India have clear indicators that represent disease burden and their trends [106]. The learnings from these can inform strategies for better reporting of cancer screening-related data. The European Commission’s initiative on breast cancer has developed 13 indicators through a rigorous 4-stage process to be implemented through the cancer continuum [107]. It also defines indicators like screening rate, coverage rate, detection rates, etc. which are used in various state reports on the NHM website. Similarly, the WHO’s global strategy to accelerate the elimination of cervical cancer has recommended the use of standard indicators to monitor screening programs and results [108]. A common platform to report is needed by using these indicators, which can monitor and evaluate programs to inform policies.

### Strengths

Our scoping review offers a comprehensive overview of the conducted breast and cervical cancer screening programs since 2016 by government as well as non-government providers. The review investigates barriers and facilitators (Table 2) of screening programs to suggest feasible solutions like involving larger groups of stakeholders, task sharing, credentialing community health workers, and bringing care closer to homes. The review also highlights the gap in reporting the data on screening by peer-reviewed articles and government reports.

### Limitations

The manuscript has a few limitations. This study focuses on literature from years 2015 onwards and may have missed the long-term trends or the important progress from the previous cancer screening program. Data from some of the studies conducted by the government tertiary care hospitals may have been documented both on government websites as well as through peer-reviewed programs. We have not isolated those manuscripts and analyzed them separately since we have not used pooled data or meta-analysis. We did not formally assess the risk of bias or the methodological quality of the included studies, which is a recognized limitation of the scoping review approach. This review selected articles restricted to English-language publications, which may have excluded relevant studies published in other languages or in non-indexed formats. In addition, this review focused solely on breast and cervical cancers and did not include other common cancers in India (e.g., oral or colorectal), potentially limiting insights into broader or integrated cancer control strategies.

## Conclusion

Screening programs are concentrated in states with higher GDPs, leaving out states with lower GDPs and low health indicators and aspirational states. The available literature lacks uniform monitoring and evaluation indicators. Literature has extensively documented initiatives and solutions to increase the participation of women in screening initiatives. However, very few manuscripts have addressed innovative approaches to improve low-cost, closer-to-home solutions for the provision of services for screening. A lot can be learned from examples of other LMICs in sub-Saharan Africa, and Central America, and global initiatives for cancer screening by the WHO to create better provision and coverage of screening in India.

## Supplementary Information

Supplementary Material 1. Supplementary Table 1 A detailed search strategy customized for six databases used for this scoping review is compiled here.

Supplementary Material 2. Supplementary Table 2 List of 59 included articles included in this review [109–117].

Supplementary Material 3. Supplementary Table 3 National Program for Prevention and Control of Non-Communicable Diseases (NP-NCD) state-level key deliverables on breast and cervical cancer screening coverage as reported under the National Health Mission (NHM).

## Data Availability

All the databases utilized for this study included open-access articles, publicly available data, and articles compiled from six databases: PubMed, Web of Science, SCOPUS, CINAHL, Embase, and Google Scholar. The datasets generated, compiled, and/or analysed for this study are provided as supplementary files in the submission system.

## Abbreviations

AIIMS: All India Institute of Medical Sciences
ASHA: Accredited Social Health Activist
CBE: Clinical Breast Examination
CHW: Community Health Worker
HICs: High-Income Countries
HPV: HumanPapillomavirus
iBE / iBreastExam: Handheld breast screening device
KAP: Knowledge, Attitude, and Practice
LMICs: Low- and Middle-Income Countries
mHealth: Mobile Health Technology
MoHFW: Ministry of Health and Family Welfare
NGO: Non-Governmental Organization
NHM: National Health Mission
NFHS-5: National Family Health Survey–5
NP-NCD: National Programfor Prevention and Control of Non-Communicable Diseases
OOPE: Out-of-Pocket Expenditure
PAP: Papanicolaou test / Pap smear
PHC: Primary Health Center
PM-JAY: Pradhan Mantri Jan Arogya Yojana
PIP: Program Implementation Plan
PRISMA: Preferred Reporting Items for Systematic Reviews and Meta-Analysis
ROP: Recording of Proceedings
RCT: Randomized Controlled Trial
RMNCH+A: Reproductive, Maternal, Newborn, Child, and Adolescent Health
UT: Union Territory
VIA: Visual Inspection with Acetic Acid
VILI: Visual Inspection with Lugol’s Iodine
WHO: World Health Organization
