# Supplementary material for: Low participation in cancer screening in India: a scoping review of breast and cervical cancer programs
Source: BMC Cancer. 2025 Nov 7;25:1724. doi: 10.1186/s12885-025-14859-6 (PMC12593890; doi:10.1186/s12885-025-14859-6)
Supplement: Supplementary file 1 — Supplementary Material 1. Supplementary Table 1 A detailed search strategy customized for six databases used for this scoping review is compiled here. [file 12885_2025_14859_MOESM1_ESM.doc]

**Supplementary Data**

**Supplementary Table 1**: A detailed search strategy customized for six databases used for this scoping review is compiled here.

| 1. 1. **PubMed:** Date accessed 7^th^ July 2023   (breast cancer [MeSH Terms]) OR (cervical cancer, uterine [MeSH Terms])) AND (mass screening [MeSH Terms])) OR (early detection of cancer [MeSH Terms])) OR (cancer screening [MeSH Terms])) AND (India) NOT (Genetics [MeSH Subheading])  Female, Adult: 19-44 years, Middle Aged: 45-64 years, from 2015 – 2023  **PubMed Search Results:** 157 documents found |
| --- |
| 1. **Web of Science:** Date accessed 7^th^ July 2023   (ALL=(Breast cancer) OR ALL=(Breast neoplasm) OR ALL=(Cervical cancer)) AND (ALL=(Screening) OR ALL=(Early detection)) AND (ALL=program OR ALL=Trial) AND (ALL=India) NOT (AB=(Guidelines) OR AB=(High-Income countries) OR AB=(burden of disease) OR AB=(Vaginal cancer))  Filters: from 2015 – 2023  **Web of Science Search Results:** 374 documents found. |
| 1. **CINAHL:** Date accessed  7^th^ July 2023   SU breast cancer OR SU cervical cancer AND mass screening OR ( early detection or early diagnosis or early identification ) OR ( cancer screening or cancer prevention ) AND India NOT genetics  Limiters - Published Date: 20150101-20231231  Narrow by Language: - English  Search modes - Boolean/Phrase  42,777  Narrow by Subject Geographic: - Asia  Narrow by Subject Gender: - Female  Narrow by Subject Age: - aged: 65+ years,  Middle-aged: 45-64 years, adult: 19-44 years  489  Expanders - Apply equivalent subjects  Narrow by Subject Major: - cytodiagnosis  Narrow by Subject Major: - clinical assessment tools  Narrow by Subject Major: - women's health  Narrow by Subject Major: - mammography  Narrow by Subject Major: - diagnostic imaging  Narrow by Subject Major: - socioeconomic factors  Narrow by Subject Major: - cervical smears  Narrow by Subject Major: - tertiary health care  Narrow by Subject Major: - cancer patients  Narrow by Subject Major: - breast self-examination  Narrow by Subject Major: - neoplasms  Narrow by Subject Major: - health knowledge  Narrow by Subject Major: - cancer screening  Narrow by Subject Major: - attitude to health  Narrow by Subject Major: - health screening  Narrow by Subject Major: - early diagnosis  Narrow by Subject Major: - cervix neoplasms  Narrow by Subject Major: - early detection of cancer  Narrow by Subject Major: - breast neoplasms  **CINAHL Search Results:** 170 documents found**.** |
| 1. **EMBASE:** Date accessed  7^th^ July 2023   (('breast cancer'/exp/mj OR 'breast cancer' OR 'breast cancer'/mj/exp OR 'breast cancer' OR 'uterine cervix cancer'/exp/mj OR 'uterine cervix cancer' OR 'uterine cervix cancer'/mj/exp OR 'uterine cervix cancer') AND ('mass screening'/exp/mj OR 'mass screening' OR 'mass screening'/mj/exp OR 'mass screening') OR 'early cancer diagnosis'/exp/mj OR 'early cancer diagnosis' OR 'early cancer diagnosis'/mj/exp OR 'early cancer diagnosis') AND ('india'/exp/mj OR 'india' OR 'india'/mj/exp OR 'india') NOT ('genetic'/exp/mj OR 'genetic' OR 'genetic'/mj/exp OR 'genetic') AND [female]/lim AND ([middle aged]/lim OR [aged]/lim) AND [humans]/lim AND [english]/lim AND [2015-2023]/py  **EMBASE Search Results:** 238 documents found. |
| 1. **Scopus Search:** Date accessed 23^rd^ May 2023   **Query:** Breast cancer OR Breast neoplasm) OR (Cervical cancer)) AND (Screening OR Early detection)) AND (program or Trial)) AND (India) AND (2015:2023[pdat]) NOT (Guidelines))) NOT (High Income countries)) NOT (burden of disease[Title/Abstract]) AND (2015:2023[pdat])) NOT (Vaginal cancer) Filters for year set again: from 2015 – 2023  Results: 1, 04,558 documents found  Revising search criteria by limiting to India  Results: 7,218 documents found  Limiting to English  Results: 7,216 documents found  Excluded Veterinary  Excluded Dentistry  Excluded Earth and Planetary Sciences  Excluded Economics, Econometrics and Finance  Excluded Neuroscience  Excluded Energy  Excluded Business, Management and Accounting  Excluded Environmental Science  Excluded Physics and Astronomy  Excluded Mathematics  Excluded Materials Science  Excluded Chemical Engineering  Excluded Chemistry  Excluded Engineering  Excluded Computer Science  Excluded Pharmacology, Toxicology and Pharmaceutics  Excluded Biochemistry, Genetics and Molecular Biology  Excluded Agricultural and Biological Sciences  Excluded Immunology and Microbiology **Scopus Search Results**: 879 documents found |
| 1. **Google Scholar:** Date accessed 17^th^ July, 2023   **With Google Scholar, a general keyword** search was done using Harzing’s Publish or Perish Software (https://harzing.com/resources/publish-or-perish) using search terms **“**Breast and cervical cancer screening programs, India, early detection, mass screening”. All articles were downloaded into a CSV file.  **Google Scholar Results:** 870 articles found. |
