# Supplementary material for: Low participation in cancer screening in India: a scoping review of breast and cervical cancer programs
Source: BMC Cancer. 2025 Nov 7;25:1724. doi: 10.1186/s12885-025-14859-6 (PMC12593890; doi:10.1186/s12885-025-14859-6)
Supplement: Supplementary file 2 — Supplementary Material 2. Supplementary Table 2 List of 59 included articles included in this review [109–117]. [file 12885_2025_14859_MOESM2_ESM.doc]

**Supplementary Table 2**. List of 59 included articles included in this review.

| **Sr No** | **Title of Paper** | **Publication Year** | **Cancer Type studied** | **Outreach Strategy** | **State/Union Territory** | **District** |
| --- | --- | --- | --- | --- | --- | --- |
| 1 | Barriers and facilitators in implementing population based common cancer screening through community health workers [68]. | 2021 | Both | Population based | Assam | Cachar |
| 2 | Screening of cancer cervix: Pap smear in rural India [109]. | 2016 | Cervical | Population-based | Tamil Nadu | Thiruvallur |
| 3 | Analysing the Insights and Assessing the Impact of a Digital Mammography and Tomosynthesis Based 2-year Long Prospective Breast Screening Programme Organised in Western India [49]. | 2022 | Breast | Hospital-based | Maharashtra | Pune |
| 4 | Cost-Effectiveness of Cervical Cancer Screening in Rural Bengaluru with Demographic Analysis of Epithelial Cell Abnormalities: A Cross-Sectional Descriptive Study [64]. | 2022 | Cervical | Population-based | Karnataka | Bangalore Rural |
| 5 | Cross-sectional study on visual inspection with acetic acid and pap smear positivity rates according to sociodemographic factors among rural married women of Bareilly (Uttar Pradesh) [59]. | 2018 | Cervical | Population-based | Uttar Pradesh | Bareilly |
| 6 | A study on cervical cancer screening in symptomatic women using Pap smear in a tertiary care hospital in rural area of Himachal Pradesh, India [43]. | 2017 | Cervical | Hospital-based | Himachal Pradesh | Kangra |
| 7 | A successful model of cancer screening in low resource settings: Findings of an Integrated Cancer Screening Camp from a Rural Setting of North India [60]. | 2020 | Both | Population-based | Haryana | Panchkula |
| 8 | Diagnostic accuracy of VIA and HPV detection as primary and sequential screening tests in a cervical cancer screening demonstration project in India [21]. | 2015 | Cervical | Population-based | West Bengal | Kolkata |
| 9^#&^ | Mobile technology and cancer screening: Lessons from rural India [19]. | 2018 | Cervical | Population-based | Tamil Nadu, Chhattisgarh, Madhya Pradesh | Vellore, Mungeli, Chhindwara |
| 10 | Cervical Cancer Screening in HIV-Positive Women in India: Why, When and How? [35]. | 2021 | Cervical | Hospital-based | Delhi | New Delhi |
| 11^#^ | Menstrual pad, a cervical cancer screening tool, a population-based study in rural India [42]. | 2017 | Cervical | Population-based | Maharashtra | Ahmednagar, Pune |
| 12 | A retrospective study on cervical cancer screening-In a newly opened tertiary care Centre in Eastern India [77]. | 2023 | Cervical | Hospital-based | West Bengal | Nadia |
| 13 | Efficacy of VIA, VILI, PAP Smear, and FRD Tests in Screening for Cervical Cancer: A Comparative Study [38]. | 2021 | Cervical | Hospital-based | Tamil Nadu | Chennai |
| 14^#^ | Diagnostic Accuracy of Conventional Cell Blocks Along with p16INK4 and Ki67 Biomarkers as Triage Tests in Resource-poor Organized Cervical Cancer Screening Programs [110]. | 2019 | Cervical | Population-based | Manipur | Different parts of Manipur (Tamenglong, Imphal East) |
| 15^#^ | Call for systematic Population-Based cervical Cancer screening: findings from Community-Based screening camps in Tamil Nadu, India [56]. | 2019 | Cervical | Population-based | Tamil Nadu | Tirunelveli, Thoothukkudi |
| 16 | Cancer early detection program based on awareness and clinical breast examination: Interim results from an urban community in Mumbai, India [53]. | 2017 | Breast | Population-based | Maharashtra | Mumbai |
| 17 | Setting up a Breast Cancer Awareness Project in Mumbai: Methodology, Experiences and Challenges [54]. | 2019 | Breast | Population-based | Maharashtra | Mumbai |
| 18 | Evaluation of cytology as secondary triage in visual inspection after application of 4% acetic acid-based cervical cancer screening program [66]. | 2019 | Cervical | Population-based | Maharashtra | Mumbai |
| 19 | Visual inspection methods as screening test for cervical cancer in low-resource settings [111]. | 2018 | Cervical | Hospital-based | West Bengal | Kolkata |
| 20 | Can financial incentives encourage women to participate in a cervical cancer screening programme? Evidence from a randomized controlled trial analysis [47]. | 2022 | Cervical | Population-based | Assam | Dibrugarh, Jorhat |
| 21 | Opportunistic cervical cancer screening of women visitors at a trade fair in India [58]. | 2017 | Cervical | Population-based | Delhi | New Delhi |
| 22 | Feasibility, uptake and real-life challenges of a rural cervical and breast cancer screening program in Vellore, Tamil Nadu, South India [30]. | 2020 | Both | Hospital-based | Tamil Nadu | Vellore |
| 23 | Mobile Screening Unit (MSU) for the Implementation of the 'Screen and Treat' Programme for Cervical Cancer Prevention In Pune, India [34]. | 2021 | Cervical | Population-based | Maharashtra | Pune |
| 24 | Compliance for Community-Based Cervical Cancer Screening Program among Women of Age 30-65 Years Residing in Low Socioeconomic Settings of Mumbai, India [63]. | 2022 | Cervical | Population-based | Maharashtra | Mumbai |
| 25^&^ | Implementation of a large-scale breast cancer early detection program in a resource-constrained setting: real-world experiences from 2 large states in India [51]. | 2022 | Breast | Population-based | Uttar Pradesh, Jharkhand |  |
| 26 | Evaluation of an interventional health education project: Screening of breast cancer and health education (SHE) [112]. | 2022 | Breast | Population-based | Uttarakhand | Tehri Garhwal |
| 27 | Experience of cervical Pap smear screening in tertiary care hospital [113]. | 2020 | Cervical | Hospital-based | Delhi | New Delhi |
| 28 | Single lifetime cytological screening in high risk women as an economical and feasible approach to control cervical cancer in developing countries like India [22]. | 2015 | Cervical | Hospital-based | Uttar Pradesh | Lucknow |
| 29 | Results of Cervical Cancer Screening in the Rural Population of Lucknow West, India, through a Camp Approach [61]. | 2018 | Cervical | Population-based | Uttar Pradesh | Lucknow |
| 30 | HPV detection-based cervical cancer screening program in low-resource setting: lessons learnt from a community-based demonstration project in India [39]. | 2016 | Cervical | Population-based | West Bengal | Kolkata |
| 31 | An observational study of screening, diagnosis, and management of operable cases of cervical cancer in a tertiary institute [28]. | 2019 | Cervical | Hospital-based | Maharashtra | Mumbai |
| 32 | A pilot study to evaluate home-based screening for the common non-communicable diseases by a dedicated cadre of community health workers in a rural setting in India [41]. | 2019 | Both | Population-based | Rajasthan | Udaipur |
| 33 | Acceptability and concordance of self-versus clinician-sampling for HPV testing among rural south Indian women [40]. | 2021 | Cervical | Population-based | Karnataka | Mysore |
| 34 | A study on cervical cancer screening using pap smear in urban area in state of Meghalaya, India [76]. | 2018 | Cervical | Hospital-based | Meghalaya | East Khasi Hills |
| 35 | Community engaged breast cancer screening program in Kannur District, Kerala, India: A ray of hope for early diagnosis and treatment [50]. | 2019 | Breast | Population-based | Kerala | Kannur |
| 36 | Impact of Health Education Intervention on Uptake of Random Screening for Cervical & Breast Cancers among Rural Women in Villages of Gurgaon [52]. | 2016 | Both | Population-based | Haryana | Gurgaon |
| 37 | Visual Inspection with Acetic Acid (VIA) Screening Program: 7 Years Experience in Early Detection of Cervical Cancer and Pre-Cancers in Rural South India [23]. | 2015 | Cervical | Population-based, Hospital-based | Andhra Pradesh* | Mahabubnagar* |
| 38 | Study of pap smear as cervical cancer screening: A prospective study at a periurban tertiary care centre [114]. | 2021 | Cervical | Hospital-based | Maharashtra | Nagpur |
| 39 | Camp Screening of Symptomatic Tribal Females for Cervical Cancer in Rural Area of Rajasthan [44]. | 2019 | Cervical | Hospital-based | Rajasthan | Dungarpur |
| 40 | Comparative Study of Smart Scope® Visual Screening Test with Naked Eye Visual Screening and Pap Test [37]. | 2020 | Cervical | Hospital-based | Maharashtra | Pune |
| 41 | Effectiveness of triennial screening with clinical breast examination: 14-years follow-up outcomes of randomized clinical trial in Trivandrum, India [67]. | 2023 | Breast | Population-based | Kerala | Trivandrum |
| 42^#^ | Impact of HPV molecular testing with partial genotyping as a feasibility study in cervical cancer community screening program in South India [57]. | 2023 | Cervical | Population-based | Tamil Nadu | Chennai, Thoothukkudi, Kanyakumari |
| 43^#^ | Experiences from cervical cancer screening program conducted at low-resource areas in Telangana [115]. | 2018 | Cervical | Population-based, Hospital-based | Telangana | Medchal–Malkajgiri, Hyderabad, Ranga Reddy, Peddapalli, Vikarabad, Karimnagar, Nagarkurnool |
| 44 | Knowledge and attitude towards, and the utilisation of cervical and breast cancer screening services by female healthcare professionals at a tertiary care hospital of Eastern India: A cross-sectional study [78]. | 2022 | Both | Hospital-based | Odisha | Khordha |
| 45 | Screening for Early Detection of Cervical Cancer in Women Living with HIV in Mumbai, India-Retrospective Cohort Study from a Tertiary Cancer Center [36]. | 2022 | Cervical | Hospital-based | Maharashtra | Mumbai |
| 46^#^ | Experience of a 'Screen and treat' program for secondary prevention of cervical cancer in Uttar Pradesh, India [46]. | 2019 | Cervical | Population-based, Hospital-based | Uttar Pradesh | Lucknow, Kanpur Nagar, Varanasi, Agra, Aligarh, Allahabad, Bareilly, Ghaziabad, Gorakhpur and Mathura |
| 47 | Cancer detection rates in a population-based, opportunistic screening model, New Delhi, India [24]. | 2015 | Both | Population-based | Delhi | New Delhi |
| 48 | Can we increase the cervical cancer screening interval with an HPV test for women living with HIV? Results of a cohort study from Maharashtra, India [45]. | 2023 | Cervical | Population-based | Maharashtra | Pune |
| 49 | Cervical Cancer Screening: A Cross-Sectional Study Conducted in Tertiary Care Center in Indore [116] . | 2023 | Cervical | Hospital-based | Madhya Pradesh | Indore |
| 50 | Noninvasive and Low-Cost Technique for Early Detection of Clinically Relevant Breast Lesions Using a Handheld Point-of-Care Medical Device (iBreastExam): Prospective Three-Arm Triple-Blinded Comparative Study [31]. | 2016 | Breast | Hospital-based | Karnataka | Bangalore |
| 51 | Community-based Mobile Cervical Cancer Screening Program in Rural India: Successes and Challenges for Implementation [48]. | 2021 | Cervical | Population-based | Karnataka | Mysore |
| 52 | Cervical cancer screening behind bars: A woman's right [75]. | 2020 | Cervical | Population-based | Delhi | New Delhi |
| 53 | Impact of health education intervention in promoting cervical cancer screening among rural women of Chengalpattu district - The community based interventional study [55]. | 2021 | Cervical | Population-based | Tamil Nadu | Chengalpattu |
| 54 | Study on Cervical Cancer Screening Using Pap Smear and Related Factors among Women in Dhiraj Hospital [29]. | 2022 | Cervical | Hospital-based | Gujarat | Vadodara |
| 55 | Effect of Screening on Variation in Cervical Cancer Survival by Socioeconomic Determinants--a Study from Rural South India [25]. | 2015 | Cervical | Population-based | Tamil Nadu | Dindigul |
| 56 | Determinants of Compliance for Breast and Cervical Cancers Screening among Female Police Personnel of Mumbai, India-A Cross-Sectional Study [62]. | 2022 | Both | Population-based | Maharashtra | Mumbai |
| 57^#^ | Cervical Cancer Care Continuum in South India: Evidence from a Community-based Screening Program [65] | 2020 | Cervical | Population-based | Tamil Nadu | Tirunelveli, Thoothukkudi |
| 58 | Referral mechanism and beneficiary adherence in cervical cancer screening program in Tiruchirappalli district, Tamil Nadu state, India, 2012-2015 [79] | 2021 | Cervical | Population-based | Tamil Nadu | Tiruchirappalli |
| 59 | A Randomized Clinical Trial of Human Papillomavirus (HPV) Test-and-Treat as Compared to Cytology-Based Screening for Prevention of Cervical Cancer Among Women Living With Human Immunodeficiency Virus (HIV): AIDS Clinical Trials Group Protocol A5282 [117] | 2022 | Cervical | Hospital-based | Maharashtra | Pune |

**Footnote for Supplementary Table 2.** *Denotes the district belonged to Andhra Pradesh administration during the study period before the split into two states Andhra Pradesh and Telangana on 2nd June 2014; ^&^ Denotes data was reported from multiple states; ^#^ Denotes data was reported from multiple districts.
